# TDP-43 safeguards the embryo genome from L1 retrotransposition

**DOI:** 10.1126/sciadv.abq3806

**Published:** 2022-11-23

**Authors:** Ten D. Li, Kensaku Murano, Tomohiro Kitano, Youjia Guo, Lumi Negishi, Haruhiko Siomi

**Affiliations:** ^1^ Department of Molecular Biology, Keio University School of Medicine, Tokyo 160-8582, Japan.; ^2^ Laboratory of Chromatin Structure and Function, Institute for Quantitative Biosciences, The University of Tokyo, Tokyo 113-0032, Japan.

## Abstract

Transposable elements (TEs) are genomic parasites that propagate within the host genome and introduce mutations. Long interspersed nuclear element-1 (LINE-1 or L1) is the major TE class, which occupies nearly 20% of the mouse genome. L1 is highly active in mammalian preimplantation embryos, posing a major threat to genome integrity, but the mechanism of stage-specific protection against L1 retrotransposition is unknown. Here, we show that TAR DNA–binding protein 43 (TDP-43), mutations in which constitute a major risk factor for amyotrophic lateral sclerosis, inhibits L1 retrotransposition in mouse embryonic stem cells (mESCs) and preimplantation embryos. Knockdown of TDP-43 resulted in massive genomic L1 expansion and impaired cell growth in preimplantation embryos and ESCs. Functional analysis demonstrated that TDP-43 interacts with L1 open reading frame 1 protein (L1 ORF1p) to mediate genomic protection, and loss of this interaction led to derepression of L1 retrotransposition. Our results identify TDP-43 as a guardian of the embryonic genome.

## Teaser

Knocking down of TDP-43 causes massive L1 retrotransposition in preimplantation embryos.

## INTRODUCTION

After fertilization, mammalian zygotes undergo preimplantation embryogenesis, during which a series of rapid and synchronous cell cycles give rise to blastocysts that are competent for implantation and development (*1*, *2*). A key step in preimplantation embryogenesis is the commencement of zygotic gene activation (ZGA) and the establishment of totipotency, which is accompanied by a burst of transposable element (TE) expression (*3*–*5*). The activation of TEs during ZGA has been hypothesized to be related to chromatin opening and early gene expression; however, TE activity poses a dire threat to genome integrity because of the random integration of these elements into previously unidentified genomic loci.

Continuous TE expansion has generated more than one-third of the mouse genome, with long interspersed nuclear element-1 (LINE-1 or L1) transposons representing the most abundant TE class. L1 elements constitute 19% of the mouse genome and propagate through a “copy and paste” genetic mechanism known as retrotransposition (*6*). More than 900,000 L1 sequences are found in the mouse genome (*7*), of which approximately 3000 are still retrotransposition competent (*8*–*10*). A retrotransposition-competent L1 consists of a 5′ untranslated region (5′UTR), two open reading frames (ORF1 and ORF2), and a 3′UTR that ends with polyadenylate (poly-A) sequence (*11*). The retrotransposition of L1 occurs via target site–primed reverse transcription (*12*). The L1 mRNA directs translation of two proteins, L1 ORF1p and L1 ORF2p, which correspond to the two ORFs, respectively (*11*). In the cytoplasm, L1 ORF1p mediates ribonucleoprotein (RNP) formation of L1 mRNA, L1 ORF1p, and L1 ORF2p through its RNA binding and molecular chaperone activities (*13*, *14*). The RNP complex is imported into the nucleus, where L1 mRNA is used as a template to generate cDNA through reverse transcriptase activity of L1 ORF2p (*15*). Last, retrotransposition is achieved by ligation of the cDNA with genomic DNA that bears a single-strand break created by the endonuclease activity of L1 ORF2p (*16*). It has been shown that some diseases including certain types of cancer, hemophilia A/B, and severe combined immunodeficiency can be caused by deleterious L1 insertions (*17*). Because of their high potential for mutagenicity, L1 loci are stringently silenced by repressive epigenetic modifications in most tissues (*18*). However, the erasure of epigenetic modifications that occurs in preimplantation embryos results in extensive L1 activation, which jeopardizes genome integrity (*4*, *18*). While preimplantation embryos are abundantly loaded with L1 RNP complexes (*5*), how they counteract L1 retrotransposition remains completely unclear.

Transactive response (TAR) DNA–binding protein 43 (TDP-43) was first identified as a transcriptional regulator that suppresses HIV-1 gene expression and protects against viral infection (*19*). Previous studies have shown that TDP-43 is an RNA binding protein with several functions including mRNA transcription, translation, splicing, and stability (*20*, *21*). Screening of amyotrophic lateral sclerosis (ALS) risk factors showed that ectopic expression of TDP-43 is associated with reduced L1 retrotransposition activity in reporter system using human embryonic kidney (HEK) 293T cells (*22*). In *Drosophila*, TDP-43 overexpression or knockout (KO) appears to impair the Dicer-2/Ago2-mediated small interfering RNA (siRNA) silencing system (*23*). However, a causality role of TDP-43 in L1 neutralization in vivo, particularly in preimplantation embryos where genomic integrity is cardinally important, has not been identified.

Here, we found that TDP-43 interacts with L1 ORF1p in mouse embryonic stem cells (mESCs) and inhibits embryonic L1 retrotransposition. Our results suggest that TDP-43 acts as a guardian against L1 exposure during preimplantation embryogenesis and safeguards genomic integrity.

## RESULTS

### TDP-43 interacts with L1 ORF1p and inhibits L1 retrotransposition

**Fig. 1.**
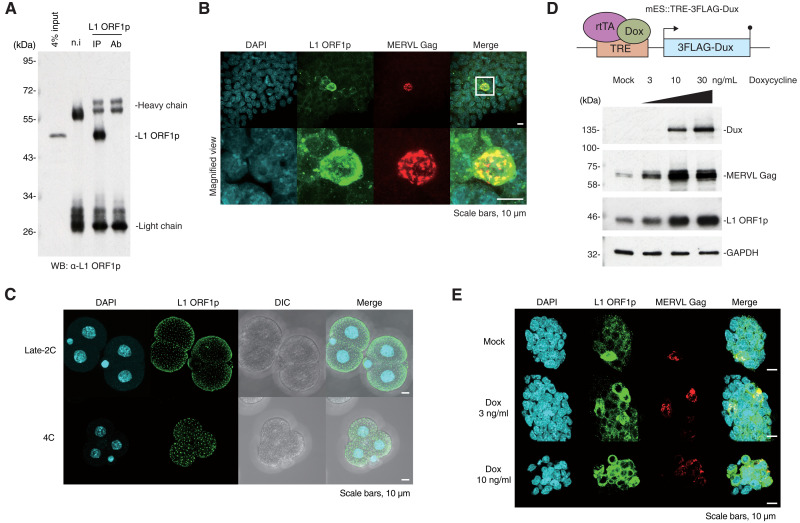
Characterization of L1 ORF1p in mESCs and mouse preimplantation embryos. (**A**) IP of endogenous L1 ORF1p in wild-type mESCs followed by WB. n.i, nonimmunized mouse [immunoglobulin G (IgG) control]; Ab, antibody only. (**B**) Immunofluorescence of wild-type mESCs shows colocalization of endogenous L1 ORF1p and MERVL Gag in 2C-like cells. Images are maximal *Z* projections of confocal sections. (**C**) Immunofluorescence of mouse embryos at late two-cell (2C) stage and four-cell (4C) stage. L1 ORF1p localized on the surface of the embryo with evenly scattered foci. Also see fig. S1B. Images are maximal *Z* projections of confocal sections. DIC, differential interference contrast microscope. (**D**) Top: Scheme of mES::TRE-3FLAG-Dux cell line construct. 3FLAG-Dux is inserted after the TRE promoter, which drives downstream gene expression upon induction by doxycycline. Bottom: MERVL Gag and L1 ORF1p are up-regulated in mES::TRE-3FLAG-Dux cell line in a doxycycline dose-dependent manner. (**E**) Immunofluorescence of mES::TRE-3FLAG-Dux cells. Images are maximal *Z* projections of confocal sections. Proportion of cells expressing L1 ORF1p and MERVL Gag were increased in a doxycycline dose-dependent manner.

We sought to characterize L1 retrotransposition inhibition during preimplantation development by identifying proteins that interact with factors required for L1 retrotransposition. L1 ORF1p is essential for L1 retrotransposition (*14*) and is highly expressed in preimplantation embryos (*5*). We raised mouse monoclonal antibodies against mouse L1 ORF1p (Fig. 1A and fig. S1A) and confirmed expression of L1 ORF1p in mESCs and preimplantation embryos (Fig.1, B and C). L1 ORF1p is evident in foci throughout the embryo and is evenly distributed near the cell membrane (Fig. 1C and fig. S1, B and C). In mESC cultures, two-cell embryo-like (2C-like) cells comprise less than 1% of the population and are a rare and transient population with totipotent features (*24*). While ESCs correspond to the inner cell mass of the blastocyst, 2C-like cells have transcriptomic profiles resembling those of 2C-stage embryos, which highly express a 2C-specific TE, mouse endogenous retrovirus with leucine transfer RNA primer (MERVL) (*24*), and L1. Immunofluorescence staining of L1 ORF1p and MERVL group-specific antigen (Gag) in mESCs showed that L1 ORF1p and MERVL Gag are both highly expressed and localize in the cytoplasm of 2C-like cells (Fig. 1B).

Dux is a transcription factor that activates 2C-specific genes during embryogenesis, and ESCs with ectopic expression of Dux acquire a 2C-like state (*25*). To assess the consequences of Dux expression on retrotransposon protein expression, we established a Dux-inducible mESC line mES::TRE-3FLAG-Dux (Fig. 1D). The expression levels of MERVL Gag and L1 ORF1p in mES::TRE-3FLAG-Dux increased with Dux expression in a dose-dependent manner upon doxycycline treatment (Fig. 1, D and E).

**Fig. 2.**
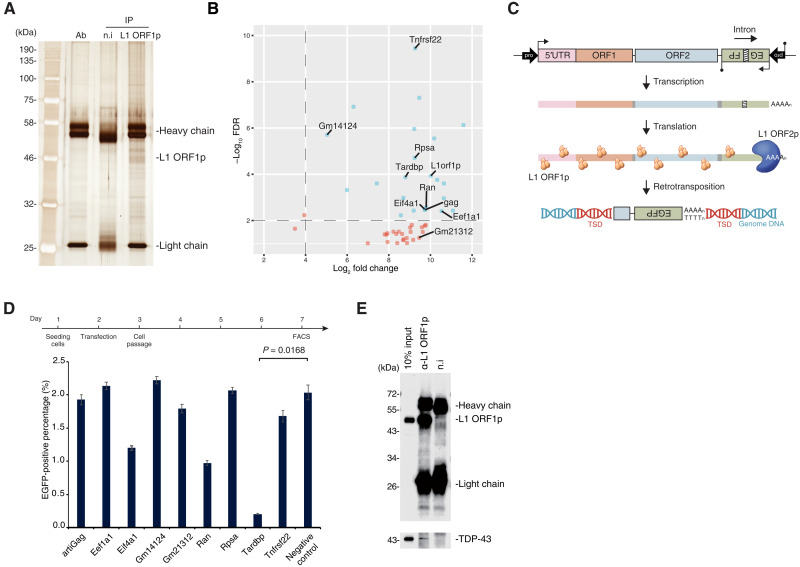
TDP-43 interacts with L1 ORF1p and inhibits L1 retrotransposition. (**A**) Silver staining of L1 ORF1p–interacting proteins co-IPed from mES::TRE-3FLAG-Dux cells after doxycycline induction. (**B**) Volcano plot showing interactome of L1 ORF1p identified by LC-MS/MS. Horizontal axis: log_2_ fold change of protein signal enrichment in anti–L1 ORF1p co-IP product versus nonimmunized IgG co-IP product; vertical axis: −log_10_ false discovery rate (FDR). Blue dots represent highly enriched proteins in the L1 ORF1p interactome. Proteins that were selected for further screening are labeled. (**C**) Scheme of the retrotransposition assay. The bivalent reporter plasmid [cep99-gfp-ORFeus-Mm (EF1αEF1α); see Materials and Methods] encodes a transposition-competent L1 followed by an antisense EGFP cassette interrupted by an intron. Once transcribed, the intron is spliced, and the mature mRNA containing an uninterrupted antisense EGFP cassette can be inserted into the host genome, leading to EGFP-positive cells. TSD, target site duplication. (**D**) Effects of L1 ORF1p interactors on L1 retrotransposition was examined by fluorescence-activated cell sorting (FACS) of HEK293T cells subjected to retrotransposition assay with ectopic expression of candidate proteins in (B). Overexpression of TDP-43 markedly repressed L1 retrotransposition. Negative control representing cells transfected with empty vector. (**E**) IP of L1 ORF1p followed by WB using mES::TRE-3FLAG-Dux lysate. Interaction of endogenous L1 ORF1p and TDP-43 was confirmed.

Next, L1 ORF1p-associated complexes were immunopurified (IP) from Dux-induced 2C-like cells (Fig. 2A) and subjected to liquid chromatography–tandem mass spectrometry (LC-MS/MS) to identify their components (table S1). As expected, L1 ORF1p was highly enriched in the IP samples. Among the identified L1 ORF1p interactome, eight highly enriched proteins were selected empirically for further investigation, and an interacting protein below our significance threshold, Gm21312, was chosen as control (Fig. 2B).

We then performed L1 retrotransposition assays (*26*, *27*) in the presence of the selected interactors to examine whether these proteins are capable of inhibiting L1 retrotransposition (Fig. 2C). Briefly, the bivalent L1 reporter plasmid encodes a transposition-competent L1 followed by an antisense enhanced green fluorescent protein (EGFP) cassette interrupted by a sense intron. Upon L1 transcription, the intron in EGFP is spliced, and the processed mRNA containing an intact antisense EGFP cassette can be reverse-transcribed and inserted into the host genome, leading to EGFP-positive cells that have undergone retrotransposition and can be detected by flow cytometry. To validate that this assay can be used to detect retrotransposition inhibition in HEK293T cells, we confirmed a dose-dependent decrease in retrotransposition frequency upon administration of tenofovir, which specifically inhibits reverse transcription (fig. S2, A and B; also see details in Materials and Methods). This retrotransposition assay was performed in HEK293T cells with ectopic expression of cDNAs encoding the selected L1 ORF1p–interacting proteins (fig. S2C). Retrotransposition frequency, as measured by the EGFP-positive cell population, was markedly decreased in cells transfected with the plasmid expressing *Tardbp*, which encodes the protein TDP-43 (Fig. 2D and fig. S2D). In contrast, overexpression of TDP-43 did not affect the splicing and expression of the reporter gene (fig. S2, E and F). Co-IP followed by Western blotting (WB) in doxycycline-treated mES::TRE-3FLAG-Dux cells (Fig. 2E) confirmed that TDP-43 is a bona fide interactor of L1 ORF1p.

### Zygotic TDP-43 knockdown leads to increased L1 retrotransposition and developmental defects

**Fig. 3.**
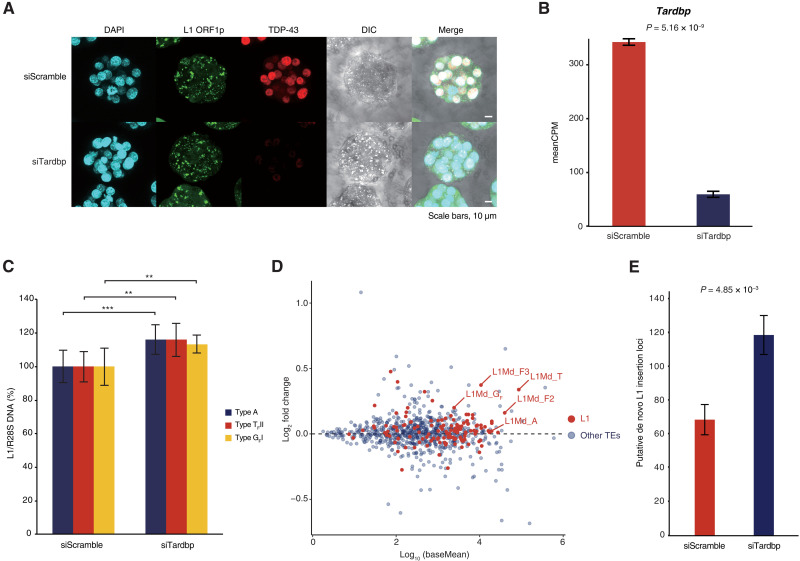
Zygotic TDP-43 KD leads to increased L1 retrotransposition. (**A**) Immunofluorescence of zygotes injected with control (siScramble) or TDP-43 (siTardbp) targeting siRNA. TDP-43 KD morulae show strongly decreased TDP-43 signal. Images are maximal *Z* projections of confocal sections. (**B**) Expression level of *Tardbp* as assessed by RNA-seq with and without KD. (**C**) qPCR using primer sets targeting active L1 subfamilies (*29*) with WGA DNA from five blastocysts (4.5 dpc) ± TDP-43 KD as template. Expression of active L1 subfamilies was increased in TDP-43 KD embryos. **P ≤ 0.01 and ***P ≤ 0.001. (**D**) MA, mean average plot showing expression change of TEs in TDP-43 KD embryos. Horizontal axis: log_10_ normalized read count (baseMean); vertical axis: log_2_ fold change of expression level in KD embryos versus control embryos. L1 elements are highlighted in red. Here, we adopted the L1 classification of repeat masker in RNA-seq analysis, so L1Md_A corresponds to subfamilies L1MdA_I, L1Md_AII, and L1Md_AIII; L1Md_T corresponds to subfamilies T_F_ and G_F_; L1Md_F2 corresponds to L1Md_AIV, L1Md_AVII, and L1Md_F; L1Md_F3 corresponds to the remaining A subfamily and partial of subfamily L1Md_N_I (*30*). (**E**) Targeted enrichment sequencing was used to detect previously unannotated putative L1 insertion sites in TDP-43 KD embryos (4.5 dpc) and in control embryos (4.5 dpc).

As we found that TDP-43 inhibits L1 retrotransposition in vitro, we next investigated its role during preimplantation development. We first analyzed previously published single-cell RNA sequencing (RNA-seq) data (*28*) to determine the preimplantation expression profiles of *Tardbp* and entire L1 family in mouse embryos (fig. S3, A and B). While *Tardbp* and L1 family are both maternally inherited, *Tardbp* transcripts are markedly depleted at the mid-2C stage before being progressively induced, whereas L1 family transcripts gradually increase after fertilization and reach their maximum level at the mid-to-late 2C stage. We raised monoclonal antibodies against TDP-43, and immunofluorescence staining of different stages of mouse embryos showed that TDP-43 is enriched in the nucleus (Fig. 3A and fig. S3C).

We then asked whether TDP-43 safeguards preimplantation embryos against L1 retrotransposition. TDP-43 knockdown (KD) was performed by microinjecting siRNA against *Tardbp* (siTardbp) into male zygote pronuclei. TDP-43 was undetectable by immunofluorescence staining in siTardbp embryos, and RNA-seq showed that *Tardbp* levels decreased to less than 20% of control morulae (siScramble; Fig. 3, A and B). Although TDP-43 KD embryos seemed to have undergone normal developmental progression at 4.5 days postcoitum (dpc) based on embryo staging (fig. S3, D and E), the volume of TDP-43 KD embryos was nearly half that of control embryos (fig. S3F), suggesting severe cell growth defects. Notably, quantitative polymerase chain reaction (qPCR) using whole genome–amplified (WGA) DNA from TDP-43 KD blastocysts (4.5 dpc) revealed significant increases in DNA amount of L1 A, G_F_, and T_F_ subfamilies (Fig. 3C), which have been reported to be evolutionarily young and retrotransposition competent (fig. S3G) (*8*–*10*, *29*, *30*). RNA-seq similarly showed that expression of active L1 was broadly up-regulated in TDP-43 KD embryos (Fig. 3D, fig. S3H, and table S2). To corroborate the findings, we performed targeted enrichment sequencing of L1 insertions by transposon insertion profiling by sequencing (TIP-seq) (*31*) using WGA DNA, which had no bias concerning amplifying β-actin gene on chromosome 5 at least (fig. S3, I and J; also see Materials and Methods). We identified an almost 70% increase in putative de novo L1 insertions in TDP-43 KD embryos (4.5 dpc) compared to controls (4.5 dpc; Fig. 3E and table S3). The raw sequence data from TIP-seq analysis showed that L1s of different origins were retrotransposed to A-rich regions on chromosomes as previously described (*11*). These loci might provide hotspots for L1 retrotransposition during preimplantation embryogenesis in the context of TDP-43 depletion (fig. S3K). A smaller number of L1 insertions unique to control embryos were also identified, suggesting a basal frequency of L1 retrotransposition that naturally occurs during embryogenesis (*32*), which may be modified by strain-specific genome sequences or whose identification may be limited by statistical power (table S4). Together, the DNA expansion and increased expression of active L1 in TDP-43 KD embryos indicate that TDP-43 is required to suppress L1 retrotransposition during early embryogenesis.

### TDP-43 mutations in mESCs result in increased L1 retrotransposition

**Fig. 4.**
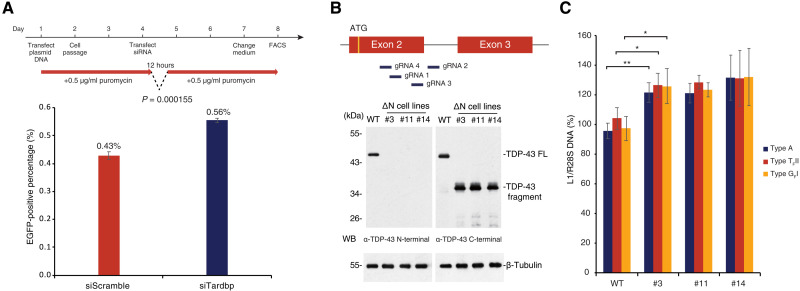
TDP-43 mutation in mESCs results in increased L1 retrotransposition. (**A**) mESCs treated with control and *Tardbp*-targeting siRNA were used for the retrotransposition assay (see Fig. 2C) and analyzed by FACS. The experimental time course is shown above. Retrotransposition frequency was increased in cells transfected with siTardbp compared with siScramble. (**B**) Strategy to KO TDP-43 using CRISPR-Cas9 with four gRNAs is illustrated in the top panel. The resulting clones are annotated as TDP-43 ΔN cell lines (#3, #11, and #14). These three monocloned lines were isolated, and N-terminal truncated TDP-43 was detected by WB using an anti–TDP-43 C-terminal antibody. Also see fig. S4 (C and D). (**C**) qPCR using primer sets targeting each active L1 subfamily (*29*) was performed on wild-type and ∆N lines. The DNA amount of active L1 subfamilies was increased in TDP-43 ΔN mESCs. *P ≤ 0.05 and **P ≤ 0.01.

That TDP-43 KO causes embryonic lethality (*33*) prevents investigation of effects of prolonged TDP-43 depletion on L1 retrotransposition in vivo, so we next asked whether TDP-43 is also responsible for inhibiting L1 retrotransposition in mESCs, which recapitulate preimplantation embryos and are readily amendable to genetic manipulation. We confirmed that endogenous TDP-43 is abundantly expressed in mESCs and can be transiently knocked down using siRNA against *Tardbp* (siTardbp; fig. S4A). We performed the retrotransposition assay in mESCs subjected to TDP-43 KD and found roughly 30% increased retrotransposition frequency, while TDP-43 KD did not affect the splicing of the reporter gene (Fig. 4A and fig. S4B). We then attempted to investigate the consequences of prolonged TDP-43 removal on L1 retrotransposition by KO TDP-43 in mESCs using CRISPR-Cas9. Four guide RNAs (gRNAs) targeting the area just downstream of the start codon of *Tardbp* (Fig. 4B) were designed and cloned into expression plasmids with Cas9 and a puromycin resistance cassette. mESCs were transfected with the plasmids and subjected to puromycin selection, resulting in three clones (#3, #11, and #14) with decreased growth rates compared to wild-type mESCs (fig. S4C). Genotyping showed that instead of complete KO, TDP-43 in these clones lacks the first 84 amino acids because of exon 2 skipping and is instead translated from an alternative start codon in exon 3 (fig. S4D), resulting in a TDP-43 ΔN mutant (Fig. 4B). These three mutant clones all have identical mRNA sequence but different genomic DNA sequences (fig. S4D). The cDNA of ΔN mutant was cloned and overexpressed in mESCs and confirmed to be the same size as the product detected in ΔN mutant cell lines (fig. S4E). Despite the continued presence of the truncated TDP-43 protein, the DNA amount of active L1 subfamilies increased by around 20 to 40% in TDP-43 ΔN mutant clones (Fig. 4C). Moreover, we performed immunofluorescence staining of wild-type mESCs and ΔN mutant cell lines and found that the fluorescence signal of L1 ORF1p is significantly higher in the nucleus (fig. S4F), with a concomitant increase in L1 ORF1p expression (fig. S4G). TDP-43 contains a bipartite nuclear localization signal (NLS) domain (81 to 87 amino acids and 94 to 100 amino acids) (*34*). The ΔN mutant of TDP-43 localized to the nucleus despite lacking a part of the domain (fig. S4, D and F), indicating that the remaining NLS domain might be exposed and functional in the ΔN mutant. In addition to confirming that TDP-43 inhibits L1 retrotransposition in mESCs, these results suggest that the N-terminal domain of TDP-43 is important for this function.

### Interaction with L1 ORF1p is required for TDP-43–mediated L1 retrotransposition inhibition

**Fig. 5.**
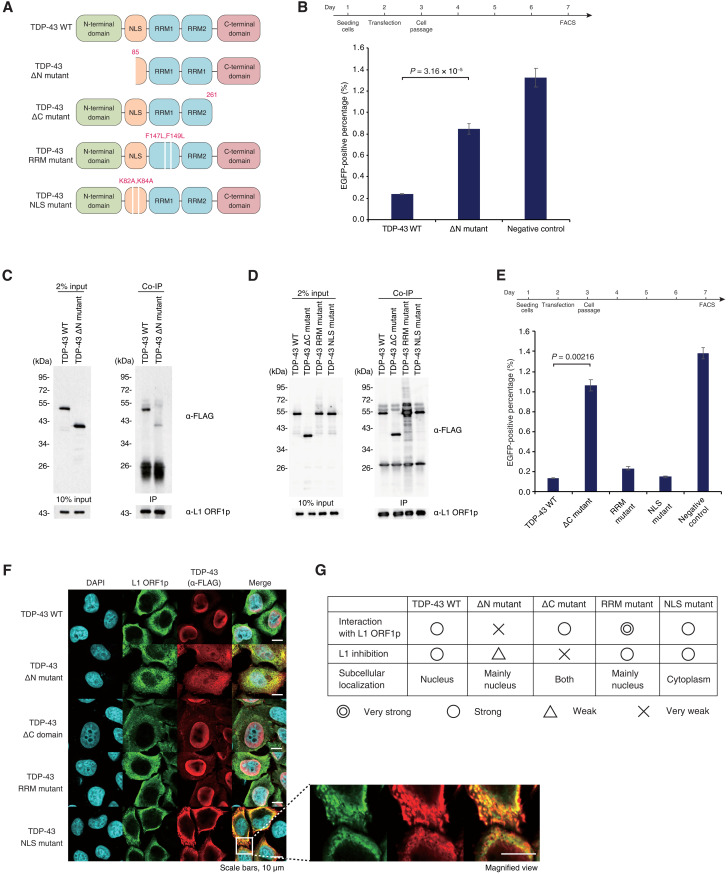
Interaction with L1 ORF1p is required for TDP-43–mediated L1 retrotransposition. (**A**) Illustration of TDP-43 mutants used in this study. (**B**) The FACS-based retrotransposition assay (see Fig. 2C) showed that retrotransposition frequency was higher in HEK293T cells with ectopic expression of the TDP-43 ΔN mutant compared to the full-length TDP-43. The experimental time course is shown in the top panel. (**C**) The interaction between the FLAG-tagged TDP-43 ΔN mutant and L1 ORF1p was examined by co-IP of L1 ORF1p in HEK293T cells. The interaction between TDP-43 ΔN mutant and L1 ORF1p was compromised relative to wild-type TDP-43. (**D**) The interaction between FLAG-tagged TDP-43 mutants (A) and L1 ORF1p was examined by co-IP of L1 ORF1p in HEK293T cells. Loss of either the C-terminal domain or mutation of the NLS did not affect TDP-43’s interaction with L1 ORF1p. (**E**) L1 retrotransposition frequency in HEK293T cells overexpressing TDP-43 mutants. The experimental time course is shown above. Inhibition of retrotransposition by TDP-43 was compromised by loss of the C-terminal domain but not other mutations. (**F**) Subcellular localization of L1 ORF1p and TDP-43 mutants in HeLa cells by immunofluorescence staining. The TDP-43 NLS mutant was localized to the cytoplasm, with significant overlap with L1 ORF1p. (**G**) Summary table of the characteristics of TDP-43 mutants.

We then sought to unveil the structural basis of TDP-43–mediated L1 retrotransposition inhibition. TDP-43 consists of an N-terminal domain that contributes to homopolymer formation, an NLS domain, two RNA recognition motif (RRM) domains, and a disordered C-terminal domain, which harbors most ALS-associated mutations that map to the gene (Fig. 5A) (*35*, *36*). As the TDP-43 ΔN mutant in mESCs causes derepression of L1 retrotransposition, we first asked whether the N-terminal domain of TDP-43 mediates its interaction with L1 ORF1p. As expected, the ability of TDP-43 expression to inhibit L1 retrotransposition was impaired in the ΔN mutant (Fig. 5B and fig. S5A), with a corresponding decrease in enrichment of the TDP-43 ΔN mutant in the L1 ORF1p co-IP compared to wild-type TDP-43 (Fig. 5C). We also confirmed that the interaction between L1 ORF1p and TDP-43 is independent of the presence of RNA (fig. S5B). We then sought to identify the functional domain responsible for inhibiting L1 retrotransposition using three TDP-43 mutants: ΔC mutant deleted for amino acids 262 to 414, RRM mutant with F147/149L substitutions that have been shown to compromise RNA binding (*35*), and NLS mutant with K82/84A substitutions (*22*) that impairs its unclear localization in the context of the full-length protein (Fig. 5A). Co-IP experiments in HEK293T cells showed that the RRM mutant was vastly enriched for binding to L1 ORF1p, while no significant change in enrichment of the ΔC or NLS mutants was observed (Fig. 5D). The L1 retrotransposition assay in HEK293T cells revealed that deletion of the C-terminal domain severely compromised the ability of TDP-43 to inhibit L1 retrotransposition, while the RRM mutant and the NLS mutant maintained their inhibitory capacity (Fig. 5E and fig. S5C). Consistent with our results in mESCs and mouse embryos, wild-type TDP-43 was localized to the nucleus, and L1 ORF1p was found throughout the cytoplasm (Fig. 5F); as expected, the NLS mutant failed to enter the nucleus and instead colocalized with L1 ORF1p in the cytoplasm. Both wild-type and NLS mutant TDP-43 repressed L1 retrotransposition effectively, and no correlation between steady-state subcellular localization and L1 inhibition ability was observed (Fig. 5, E and F). Together, these results indicate that the N-terminal domain of TDP-43 mediates its interaction with L1 ORF1p and plays an important role in L1 retrotransposition inhibition, and the C-terminal domain of TDP-43 is critical only for repressing L1 retrotransposition (Fig. 5G). These results also suggest that steady-state subcellular location of TDP-43 may not be critical for L1 repression as far as it interacts with L1 ORF1p.

## DISCUSSION

Preimplantation embryogenesis and gametogenesis are the two major reprogramming events of the mammalian life cycle (*18*, *37*). These events are accompanied by “bursts” of TE expression (*3*, *37*–*39*). While it has been established that primordial germ cells secure genome integrity by exploiting the P-element induced wimpy testis (PIWI)-PIWI-interacting RNA pathway to repress TEs (*40*), it has remained unknown how the embryonic TE burst is inhibited, especially during the earliest preimplantation stages. We have addressed this fundamental question by finding TDP-43–mediated L1 retrotransposition inhibition in mouse preimplantation embryos (Figs. 1 to 3). Our data show that the C-terminal domain of TDP-43 is essential for this function and that the N-terminal domain of TDP-43 is required for its interaction with L1 ORF1p (Fig. 5). We found that DNA amounts of active L1 subfamilies increased in mESCs endogenously expressing TDP-43 ΔN mutant protein, with a concomitant increase in L1 ORF1p expression (Fig. 4 and fig. S4). Our results suggest a model in which TDP-43 safeguards the embryonic genome by intercepting L1 RNP complexes approaching the chromosome.

Although most of the retrotransposons are severely truncated or silenced, we showed that L1 is transposition-competent during early stages of embryogenesis. Evidently, we have observed a marked increase in genomic-integrated L1 copy numbers upon TDP-43 KD (Fig. 3E and fig. S3K). However, the possibilities that the increase of L1 DNA may come from cytoplasmic cDNA, episomal cDNA circles, or RNA/DNA hybrids stalled after first strand synthesis (*41*) cannot be excluded. Accumulation of cytoplasmic L1 cDNA intermediates may trigger cyclic GMP-AMP synthase-stimulator of interferon genes activity (*42*), leading to an inflammatory response, which may result in reduced size of blastocyst (fig. S3F). There is growing evidence implicating that type I interferon response can be stimulated by increasing of cytoplasmic L1 cDNA in age-associated diseases (*43*). Moreover, in Aicardi-Goutières syndrome, an exonuclease Trex1-deficient disease, elevated L1-derived single-stranded DNA level also contributes to abnormal activation of immune response (*44*). Given that the last step of retrotransposition is speculated to occur within the nucleus, the transport mechanism of the cDNA intermediates to the cytoplasm remains unclear.

TDP-43 is a highly conserved and ubiquitously expressed protein that belongs to the heterogeneous nuclear RNP family (*45*). TDP-43 is an RNA binding protein with several functions including mRNA transcription, translation, splicing, and stability (*20*, *21*). As shown in figs. S2E and S4B, KD/overexpression of TDP-43 did not affect the splicing and expression of the reporter gene, suggesting that TDP-43 does not suppress L1 retrotransposition via splicing and translation during embryogenesis. Loss of nuclear TDP-43 has been reported to be associated with chromatin decondensation around L1 loci and increased L1 DNA content in the context of neuropathology, suggesting that TDP-43 promotes heterochromatin formation around L1 loci and represses L1 transcription (*46*). However, the heterochromatin-mediated transcriptional silencing is an unlikely mechanism of L1 repression because L1 is highly transcribed in preimplantation embryos. At this stage, there must be a posttranscriptional repression mechanism rather than pretranscriptional repression by heterochromatinization.

Mutations of TDP-43 have been found to be highly associated with ALS (*36*). Although ALS is frequently associated with elevated L1 activity (*47*, *48*), the causal relationship among TDP-43 mutations, L1 retrotransposition, and ALS pathology is under debate (*22*, *23*, *47*, *48*). ALS-associated mutations in TDP-43 are highly enriched in its C-terminal domain (*36*), which is critical for L1 retrotransposition inhibition. However, most mutations had no significant effect on the reporter gene assay in HEK293T cells (*22*). Our findings that TDP-43 deficiency leads to massive L1 retrotransposition and severely impairs embryonic growth suggest a model in which ALS pathology may be the consequence of cumulative L1 retrotransposition caused by TDP-43 dysfunction over time. The impaired mESC growth rate and reduced blastocyst size upon TDP-43 depletion may be consequences of genome instability caused by massive L1 retrotransposition, although TDP-43 is a multifunctional protein. It was previously found that TDP-43 KO embryos fail to develop beyond 8.5 dpc (*33*). Whether the expansion of L1 causes embryonic lethality in TDP-43 KO embryos remain to be investigated, as does its direct role in ALS pathology.

We have confirmed that the interaction between TDP-43 and L1 ORF1p is critical for retrotransposition inhibition, but the exact mechanism is unclear. It remains to be determined whether TDP-43 can inhibit the enzymatic activities of L1 ORF2p, physically insulates L1 RNP from approaching the chromosome, or promotes the degradative processing of L1 RNA.

## MATERIALS AND METHODS

### LC-MS/MS data

See table S1.

### RNA-seq data of mouse embryos

See table S2.

### Somatic L1 coverage of TIP-seq

See table S3.

### Germline L1 coverage of TIP-seq

See table S4.

### Plasmids used in this study

See table S5.

### PCR primers used in this study

See table S6.

### Method details

#### Monoclonal antibody production

Eight-week-old female BALB/c mice were immunized every 2 weeks for a total of six times and then boosted twice in a week. Fifty micrograms of antigen was prepared with an equal volume of TiterMax Gold adjuvant (Sigma-Aldrich) according to the manufacturers’ instructions. Four days after boosting, the splenocytes of immunized mice were collected and fused with SP2/O myeloma using electro cell fusion generator ECFG21 (Nepa Gene) according to the manufacturers’ instructions. The fused cells were cultured in GIT/IL-6/HT supplements and aminopterin medium [GIT medium (FUJIFILM Wako) supplemented with recombinant human interleukin-6 (IL-6) (1 ng/ml; PeproTech), hypoxanthine thymidine (HT) supplement (Gibco), and 0.4 μM aminopterin (Sigma-Aldrich)] for 1 week to select hybridomas. We performed enzyme-linked immunosorbent assay, WB, and IP to screen hybridomas using culture supernatant. Serial dilution was performed to monoclonize selected hybridomas. Monoclonal hybridomas were cultured in GIT medium (FUJIFILM Wako) supplemented with IL-6 (1 ng/ml) for antibody production. The isotype of antibodies was determined using the IsoStrip Mouse Monoclonal Antibody Isotyping Kit (Roche). The animal experiments were approved by the Animal Care and Use Committee of Keio University and were conducted in compliance with the Keio University Code of Research Ethics.

#### Cell culture

SP2/O myeloma and primary clones were cultured in GIT medium (FUJIFILM Wako) supplemented with IL-6 (1 ng/ml; PeproTech) under 5% CO_2_ at 37°C. The cells were subcultured every day to maintain cell density at 0.2 × 10^6^ to 1.0 × 10^6^ cells/ml. For monoclonal antibody production, hybridomas were cultured until overconfluent. The supernatants of monoclonal hybridomas were sterilized using 0.22-μm pore filters (Corning) and used directly as antibody solution in other assays.

HEK293T cells were cultured in Dulbecco’s modified Eagle’s medium (DMEM) (high-glucose; Nacalai Tesque) supplemented with 10% fetal bovine serum (FBS; Biowest), 1× GlutaMAX (Gibco), 1× sodium pyruvate (Merck), and 50 μM 2-mercaptoethanol (Gibco). Cells (5 × 10^5^) were seeded into 60-mm culture dish without coating, cultured under 5% CO_2_ at 37°C. Cells were subcultured every 3 days.

HeLa cells were cultured in DMEM (high-glucose; Nacalai Tesque) supplemented with 10% FBS, 1× GlutaMAX, 1× sodium pyruvate, and 50 μM 2-mercaptoethanol. Cells (5 × 10^5^) were seeded into 60-mm culture dish without coating, cultured under 5% CO_2_ at 37°C. Cells were subcultured every 3 days.

EB3 mESCs were cultured in DMEM (high-glucose; Nacalai Tesque) supplemented with 10% FBS, 1× GlutaMAX, 1× sodium pyruvate, 50 μM 2-mercaptoethanol, in-house–produced mouse leukemia inhibitory factor, 1 μM PD0325901 (FUJIFILM Wako), and 3 μM CHIR99021 (FUJIFILM Wako). mESCs (1 × 10^5^) were seeded into iMatrix-511 silk (Matrixome)–precoated 35-mm culture dish, cultured under 5% CO_2_ at 37°C. Cells were subcultured every 3 days.

#### Generation of transgenic mESC lines

The doxycycline-controlled mES::TRE-3FLAG-Dux cell line was generated by cotransfecting EB3 mESCs with pPB-TRE-3FLAG-Dux, pPB-CAG-rtTA3G, and pCMV-HyPBase plasmids as described previously (*49*). Forty-eight hours after transfection, the cells were subjected to hygromycin (500 μg/ml; FUJIFILM Wako) and G418 (500 μg/ml; FUJIFILM Wako) selection for 7 days. The selected cells were then seeded at 2 × 10^2^ cells/cm^2^ in a culture medium containing hygromycin (250 μg/ml) and G418 (250 μg/ml). Single-cell clones were picked and expanded after 7 days.

The TDP-43 ΔN mutant mESC lines were generated by cotransfecting EB3 mESCs with pX330-puro-Tardbp-gRNA1, pX330-puro-Tardbp-gRNA2, pX330-puro-Tardbp-gRNA3, and pX330-puro-Tardbp-gRNA4 plasmids. After 24 hours, cells were passaged and subjected to puromycin (0.75 μg/ml; Merck) selection for 4 days. The selected cells were then seeded at 50 cells/cm^2^ in culture medium containing puromycin (0.75 μg/ml). Single-cell clones were picked and expanded after 7 days.

#### siRNA transfection

mESCs were trypsinized and washed with 1× PBS (Nacalai Tesque) once. Cells (2 × 10^5^) were transfected with 40 pmol of siRNA and 20 μl of the P3 Primary Cell Nucleofector Solution (Lonza; supplement 1 added) using program CG-104 in 96-well Shuttle Device (Lonza) according to the manufacturers’ instructions. Transfected cells were then seeded into iMatrix-511 silk–precoated culture dish for culture and further experiments.

#### Immunopurification and WB

Objective culture cells were trypsinized and washed with 1× PBS once. Appropriate number of cells (1 × 10^4^ cells/μl for final lysate concentration) were resuspended with IP buffer [20 mM tris-HCl (pH 7.4), 150 mM NaCl, and 0.1% NP-40], sonicated by Bioruptor II (BM Equipment) with a total of 5 min of ON time in HIGH mode. The lysed cell solution was centrifuged at 17,700*g* for 2 min at 4°C; the supernatant was then collected as cell lysate for IP. One hundred microliters of antibodies (culture supernatant) was conjugated to 10 μl of Dynabeads Protein G (Thermo Fisher Scientific) for 30 min at 4°C, followed by washing once in IP buffer. Antibody-conjugated beads were incubated with an appropriate amount of cell lysate for 2 hours at 4°C. Beads were washed three times in IP buffer and eluted with SDS-loading dye at 95°C for 3 min. The eluted interactome was resolved on SDS–polyacrylamide gel electrophoresis and transferred onto a nitrocellulose membrane (Amersham Protran, GE Healthcare). The membrane was rinsed in PBS-T (0.1% Tween-20) three times, blocked in 2% nonfat skim milk, and then incubated in diluted primary antibody for 1 hour at room temperature. After three washes in PBS-T, the membrane was incubated in 1/5000 dilution of the peroxidase-conjugated sheep anti-mouse immunoglobulin G (IgG) secondary antibody (MP Biomedicals) for 30 min at room temperature. The membrane was washed in PBS-T three times, and signal was detected using ECL Western Blotting Detection Reagents (GE Healthcare).

#### Shotgun mass spectrometric analysis

Co-IP of L1 ORF1p was performed using mES::TRE-3FLAG-Dux lysate [induced with doxycycline (10 ng/ml) for 20 hours] with/without antibodies cross-linked to beads by 0.5% formaldehyde (Sigma-Aldrich). Immunoprecipitation using nonimmunized mouse IgG (Immuno-Biological Laboratories) was also performed as a negative control. The immunoprecipitants were eluted in elution buffer containing 10 mM tris-HCl (Nacalai Tesque) and 1% SDS (FUJIFILM Wako) by heating for 3 min at 95°C. The elutions were precipitated by trichloroacetic acid/acetone precipitation. After alkylation in iodoacetamide solution for 1 hour at room temperature with shielding from light, the proteins were concentrated by chloroform/methanol precipitation and then digested using Trypsin Gold (Promega) at 37°C overnight. An LTQ-Orbitrap Velos mass spectrometer (Thermo Fisher Scientific) equipped with a nanoLC interface (AMR, inc) was used for peptide separation and identification. The data were compared against the UniProt protein sequence database of *Mus musculus* using protein identification in the search program Proteome Discoverer 1.4 (Thermo Fisher Scientific). The *P* value of the sum posterior error probability (PEP) scores relative to negative controls was calculated using the Student’s *t* test, and then the *q* value was calculated by the Benjamini-Hochberg procedure. Only proteins detected in all three replicate experiments were used. The fold change was calculated by dividing the mean value of the sum PEP score + 1 by the value of the negative-control sum PEP score + 1. To screen candidates for L1 ORF1p interactors, proteins with a higher than 16-fold change and *q* value of <0.01 were listed as candidates.

#### L1 retrotransposition assay

L1 retrotransposition assays were performed as described previously with some modifications (*26*, *27*). cep99-gfp-ORFeus-Mm (EF1αEF1α) was used as the L1 reporter in this study. This reporter plasmid was based on cep99-gfp-ORFeus-Mm [cep99-gfp-L1SM in (*50*)] with EF1α promoters inserted into the upstream 5′UTRs of the L1 cassette and EGFP cassette for powerful expression in mESCs. To measure retrotransposition efficiency in HEK293T cells, 5 × 10^5^ cells were seeded into 0.001% poly-l-lysine (Nacalai Tesque)–precoated six-well plates and then cultured at 37°C overnight. The following day (day 2), cells were transfected with total of 2 μg of plasmid DNA using 5 μl of Lipofectamine 2000 transfection reagent (Thermo Fisher Scientific) and 250 μl of Opti-MEM (Gibco) according to the manufacturers’ instructions. The following day (day 3), transfected cells were trypsinized, and 1.5 × 10^5^ cells were passaged into each 60-mm culture dish with 0.001% poly-l-lysine coating and cultured at 37°C until day 7 without medium change. On day 7, cells were collected and resuspended in FluoroBrite DMEM (Gibco) supplemented with 10% FBS, and the proportion of EGFP-positive cells was measured using a flow cytometer (SONY SH800Z). In the established L1 retrotransposition assay, cells are typically puromycin selected after transfection with the L1 reporter to concentrate episomal L1 reporter–expressing cells. However, in our hands, administration of puromycin led to extensive cell death with overexpression of TDP-43, so we conducted the retrotransposition assay without puromycin selection, which resulted in 1 to 2% of EGFP-positive cells consistently in baseline conditions.

For mESCs, 100 μl of 2.0 × 10^5^ cell suspension was mixed with total 1 μg of plasmid DNA using 2.5 μl of Lipofectamine 2000 transfection reagent (Thermo Fisher Scientific) and 50 μl of Opti-MEM (Gibco) according to the manufacturers’ instructions. Cell-DNA mixture was then seeded into iMatrix-511 silk–precoated 96-well plate, cultured at 37°C for 6 hours, and then replaced with fresh embryonic stem cell (ES) medium. The following day (day 2), transfected cells were trypsinized, and 2.0 × 10^5^ cells were passaged into iMatrix-511 silk–precoated 35-mm culture dish with puromycin (0.5 μg/ml) (Sigma-Aldrich) ES medium. Cells were cultured at 37°C until day 5, when the medium was replaced with puromycin (0.5 μg/ml) ES medium. On day 7, cells were collected and resuspended in FluoroBrite DMEM (Gibco) supplemented with 10% FBS, and the proportion of EGFP-positive cells was measured by flow cytometry (SONY SH800Z).

#### Immunofluorescence staining

Cells were seeded on cover glasses (precoating cover glasses if need) in corresponding medium and transfected with plasmid DNAs the following day. Cells were fixed with 4% formaldehyde in PBS-T for 30 min at room temperature 48 hours after transfection. Fixed cells were washed once in PBS-T and permeabilized with 0.1% Triton X-100 (Bio-Rad) in PBS-T for 30 min at room temperature. Cells were blocked using 1% bovine serum albumin (BSA) (Sigma-Aldrich) in PBS-T for 30 min and then incubated with diluted antibody for 1 hour at room temperature. After three washes in PBS-T, cells were incubated in 1/1000 diluted Alexa Fluor 488– or Alexa Fluor 555–conjugated goat anti-mouse IgG secondary antibody (Thermo Fisher Scientific) and 4′,6-diamidino-2-phenylindole (DAPI) solution (1 μg/ml) for 30 min at room temperature in the dark. The cover glasses were mounted with the Prolong Glass Antifade Mountant (Thermo Fisher Scientific) overnight at room temperature before observing. Fluorescence images were taken with an Olympus FV3000 confocal laser scanning microscope.

For immunofluorescence staining in mouse embryos, embryos were collected after mating from 8-week-old female B6D2F1 mice injected with 150 μl of CARD HyperOva (KYUDO) and 5 IU of human chorionic gonadotropin (hCG) (ASKA Animal Health). Embryos were transferred into the EmbryoMax Advanced KSOM Embryo Medium (KSOM medium) (Sigma-Aldrich) supplemented with hyaluronidase (0.3 μg/μl) (Sigma-Aldrich) and then cultured in KSOM medium at 37°C until they developed to the desired stages. Developed embryos were treated with EmbryoMax Acidic Tyrode’s solution (Merck) to remove zona pellucida (ZP) and then fixed in 4% paraformaldehyde (Nacalai Tesque) in PBS. Fixed embryos were washed in PBS three times and permeabilized with 0.1% Triton X-100 in PBS for 20 min at room temperature. Embryos were washed three times and then blocked in 2% BSA (Sigma-Aldrich) in PBS for 20 min at room temperature. Blocked embryos were incubated with diluted antibody in 2% BSA in PBS at 4°C overnight. After three washes in PBS, embryos were transferred into 1/500 diluted Alexa Fluor 488– or Alexa Fluor 555–conjugated goat anti-mouse IgG secondary antibody (Thermo Fisher Scientific) and 1/200 diluted DAPI solution (Nacalai Tesque) and incubated for 1 hour at room temperature in the dark. Embryos were washed with PBS three times and then transferred to a clean PBS drop in a 35-mm dish with a glass bottom (Matsunami Glass), covered with paraffin liquid (Nacalai Tesque). Fluorescence images were taken with an Olympus FV3000 confocal laser scanning microscope. The animal experiments were approved by the Animal Care and Use Committee of Keio University and were conducted in compliance with the Keio University Code of Research Ethics.

#### RNA isolation and cDNA synthesis

Total RNA was isolated using ISOGEN (Nippon Gene) according to the manufacturers’ instructions. Total RNA was stored at −80°C. cDNAs were prepared using the Transcriptor First Strand cDNA Synthesis Kit (Roche) according to the manufacturers’ instructions, and the synthesized cDNAs were stored at −20°C.

#### Whole-genome amplification

Mouse embryos were collected after mating from 8-week-old female B6D2F1 mice injected with 150 μl of CARD HyperOva (KYUDO) and 5 IU of hCG (ASKA Animal Health). Embryos were transferred into KSOM medium (Sigma-Aldrich) supplemented with hyaluronidase (0.3 μg/μl) (Sigma-Aldrich) and then cultured in KSOM medium at 37°C. Microinjection was performed at 0.5 dpc under a phase-contrast inverted microscope (IX73, Olympus) equipped with a micromanipulation system (Narishige). Each siRNA (20 μM) was microinjected into the male pronuclei of zygotes using FemtoJet 4i (Eppendorf). Injected embryos were cultured in KSOM until they developed to blastocysts (4.5 dpc), which were then treated with EmbryoMax Acidic Tyrode’s solution (Merck) to remove ZP. Five siScramble-injected or five siTardbp-injected blastocysts were collected, and genomic DNA was amplified using the REPLI-g Single Cell Kit (QIAGEN) according to the manufacturers’ instructions. Three biological replicates were generated for each sample. Amplified genomic DNA was used as template for qPCR and TIP-seq to detect de novo L1 insertions. The animal experiments were approved by the Animal Care and Use Committee of Keio University and were conducted in compliance with the Keio University Code of Research Ethics.

#### Genomic DNA preparation and qPCR

Genomic DNA isolation was started with 1.0 × 10^6^ cells. Freshly harvested cells were washed with PBS once and then suspended in 500 μl of protease K buffer [1× standard saline citrate, 20 mM tris-HCl (pH 7.9), 1 mM EDTA, and 1% SDS]. Cell pellets were disrupted using a syringe to lyse cells completely. Ten microliters of protease K (20 mg/ml) (FUJIFILM Wako) was added to the lysed cell solution and incubated at 55°C for at least 2 hours. One microliter of ribonuclease A (RNase A) (10 mg/ml) (Nacalai Tesque) was added to the solution and incubated for an hour at 37°C. Genomic DNA was extracted twice by adding an equal volume of phenol/chloroform/isoamyl alcohol (25:24:1) (Nippon Gene) and then adding an equal volume of isopropanol (FUJIFILM Wako) to precipitate genomic DNA. Centrifugation at 17,700*g* for 12 min at 4°C was followed by removal of the supernatant and washing of the DNA pellet with ice-cold 70% ethanol (FUJIFILM Wako). DNA was left at room temperature for 5 min to allow the remaining water to evaporate, and 100 μl of TE (10 mM tris and 1 mM EDTA) was added to dissolve genomic DNA. One microliter of RNase A (1 mg/ml) (Nacalai Tesque) was added to the genomic DNA solution and incubated at 37°C for at least 3 hours. The solution volume was adjusted to 500 μl with protease K buffer and 3 μl of protease K (20 mg/ml) and incubated at 55°C for an hour. Phenol/chloroform/isoamyl alcohol extraction was repeated twice, adding isopropanol to precipitate genomic DNA and centrifuging as above, followed by washing the DNA pellet with ice-cold 70% ethanol once. Genomic DNA was left to air-dry at room temperature for no longer than 10 min and then dissolved in 100 μl of TE. DNA and RNA concentrations were measured using a Qubit fluorometer (Invitrogen), and DNA was kept at 4°C for short term or −20°C for long-term storage.

qPCR was performed using the TB Green Fast qPCR Mix (TaKaRa) on the Thermal Cycler Dice Real Time System (TaKaRa) according to the manufacturers’ instructions. The primer sets used are shown in table S2. Amplification efficiency of qPCR was calculated on the basis of the slope of the standard curve. After confirming amplification efficiency values, relative quantities of DNA were used in further calculations.

#### Targeted enrichment sequencing of L1 insert junctions

TIP-seq was performed as described previously (*31*). Briefly, 10 μg of mouse genomic DNA was digested by six restriction enzymes (Ase I, Bsp HI, Hind III, Nco I, Pst I, and Psu I) separately and then ligated with vectorette adaptors. Vectorette PCR was performed with an L1 sequence-specific primer combined with adaptor-specific primers (shown in table S2). The PCR products were sheared by sonicating using Covaris S2 (M&S Instruments) with intensity of 4, 10% duty cycle, and 200 cycles per burst for 100 s per sample. The sheared DNA fragments were purified by column and then used for next-generation sequencing library construction using the NEBNext Ultra II DNA Library Prep Kit for Illumina according to the manufacturers’ instructions. The libraries were quantified with 2100 Bioanalyzer (Agilent) using the Agilent High Sensitivity DNA Kit and the Kapa Library Quantification Kit (NIPPON Genetics). Quantified libraries were pooled accordingly, and deep sequencing was performed using a MiSeq sequencer [Illumina; paired-end, 150 base pairs (bp)] and HiSeq X sequencer (Illumina; paired-end, 150 bp).

Bioinformatic analysis was performed as described in the pipeline of retrotransposon capture sequencing (RC-seq) (*26*). Briefly, L1 primer sequence was trimmed from raw sequencing reads. The trimmed reads were quality controlled using fastp v0.23.2 (*51*). Quality-controlled reads were processed by FLASH v2.2.00 (*52*) with default arguments to merge overlapping reads. Merged reads were aligned to GRCm38.p6, C57BL/6NJ, and DBA/2J reference genome using Bowtie2 v2.4.1 (*53*) with default arguments. Reads mapped to at least one reference genome, and annotated L1 loci were deemed germline origin. Germline origin reads were excluded from downstream analysis. Unmapped reads were extracted and aligned to active L1 consensus sequence using LAST v1256 (-s 2 -l 12 -d 30 -q 3 -e 30) (*54*). Reads aligned ≥53 nucleotides and >95% identical to L1 consensus sequence were retained and aligned to L1 hard-masked GRCm38.p6 reference genome using Bowtie2 v2.4.1 --very-sensitive-local mode. Genomic locations mapped by more than three reads and absent from control libraries or previously annotated L1 loci were deemed somatic insertions.

#### Single-cell RNA-seq analysis

Raw single-cell RNA-seq data were obtained from the dataset of Deng *et al.* (GSE45719) (*28*). Raw sequencing reads were quality controlled using fastp v0.23.2. Quality-controlled reads were first merged by embryonic stages and aligned to the reference sequence of known mouse TEs using spliced transcripts alignment to a reference (STAR) v2.7.9a with default arguments; reads per kilobase per million mapped reads (RPKM)–normalized read coverage of active L1 subfamilies was calculated using deepTools v3.5.1 (*55*), bamCoverage function (fig. S3B). Quality-controlled reads were then aligned to reference sequence of know mouse TEs using STAR v2.7.9a (*56*) with default arguments. Reads were counted against GRCm38.p6 comprehensive gene annotation (*57*) and mm10 repeats from the University of California, Santa Cruz (UCSC) RepeatMasker annotation using Subread v2.0.1 (*58*), featureCounts function. Multimapping reads were discarded for non-TE features and counted fractionally for TEs. Counts on TE loci that belong to same subfamily were combined for downstream analysis. Seurat v4.1.0 (*59*) was used to process the read counts of single-cell RNA-seq. Cells with more than 7.5% mitochondrial reads or less than 14,000 annotated features were discarded. Expression levels were log-normalized.

#### RNA sequencing

Preparation of total RNA-seq library was performed using the SMART-Seq Stranded Kit (Clontech), according to the manufacturers’ instruction. Briefly, 19 of siScramble-injected or 23 of siTardbp-injected ZP-free embryos were lysed in 1× lysis buffer containing an RNase inhibitor (0.2 IU/μl; from SMART-Seq Stranded Kit, Clontech) directly. RNAs were sheared by heating at 85°C for 8 min and used for reverse transcription with random hexamers and PCR amplification. Ribosomal fragments were depleted from each cDNA sample with scZapR and scR-Probes. Indexed total RNA-seq libraries were enriched by second PCR amplification and then sequenced using the HiSeq X sequencer (Illumina; paired-end, 150 bp). Three biological replicates were generated for each sample. Raw sequencing reads were quality controlled using fastp v0.23.2. Quality-controlled reads were first aligned to the reference sequence of known mouse TEs using STAR v2.7.9a with default arguments; RPKM-normalized read coverage of active L1 subfamilies were calculated using deepTools v3.5.1 bamCoverage function (fig. S3H). Quality-controlled reads were then aligned to the GRCm38.p6 reference genome using STAR, with default arguments. Reads were counted against GRCm38.p6 comprehensive gene annotation and mm10 repeats from the UCSC RepeatMasker annotation using Subread v2.0.1 featureCounts function. Multimapping reads were discarded for non-TE features and counted fractionally for TEs. Counts on TE loci that belong to the same subfamily were combined for differential expression analysis performed by DESeq2 v1.32.0 (*60*).
